# Ghost peaks simulation and reduction in high-resolution echo-planar spectroscopic imaging with flyback readout

**DOI:** 10.1038/s41598-025-28277-y

**Published:** 2025-11-18

**Authors:** Jan Weis, Magor Babos, Ram Kumar Selvaraju

**Affiliations:** 1 Department of Medical Physics, Uppsala University Hospital, Uppsala, Sweden; 2 Mediso Medical Imaging Systems, Budapest, Hungary; 3 Department of Medicinal Chemistry, Uppsala University, Uppsala, Sweden

**Keywords:** Magnetic resonance, Echo-planar spectroscopic imaging, Flyback readout, Spectral ghost artifacts, High-spatial resolution, Micro-imaging, Biophysics, Medical research, Physics

## Abstract

The undesirable aspect of echo-planar spectroscopic imaging (EPSI) is the occurrence of Nyquist ghost spectral lines. This phenomenon reduces the intensity of each true spectral line due to energy transfer into one or more ghost peaks. It complicates spectra and decreases the signal-to-noise ratio of the genuine peaks. This study aims to simulate ghost spectral lines for EPSI with interleaved flyback gradient echo trains and to propose methods for reducing ghost peaks. Simulations utilized the fact that the spectra reconstructed from a single flyback gradient echo train do not contain ghost spectral lines. The acquired raw data were reorganized into interleaved two echo trains before processing. The simulations aimed to demonstrate the effects of temporal shifts, magnitude, and phase variations of echoes in the interleaved second echo train on the formation of ghost peaks. Iterative zero-order phase corrections were proposed to reduce ghost peaks. Spectra obtained from a water-vegetable oil phantom and a rat demonstrate that the zero-order phase corrections significantly decreased ghost peak magnitudes while increasing the magnitudes of the true peaks. The methods described for reducing ghost peaks are suitable for water-fat spectroscopy and imaging with high spatial and spectral resolution.

## Introduction

Echo planar spectroscopic imaging (EPSI) employs a gradient echo train for parallel encoding of one spatial and the spectroscopic dimension^1–3^. This technique accelerates magnetic resonance spectroscopic imaging (MRSI) by reducing the number of phase-encodings by one. The phase-encoding is required only for the remaining one or two spatial coordinates. However, the trade-off for reduced acquisition time is the degradation of signal-to-noise ratio (SNR) compared to standard MRSI, where all spatial coordinates are phase-encoded before sampling of the spectroscopic free induction decay. This occurs because the EPSI readout bandwidth (rBW) is one or two orders of magnitude larger than the spectral bandwidth (sBW). The wide range of rBW frequencies introduces noise at a significantly higher level compared to the sBW associated with standard MRSI. Consequently, the SNR per unit time and volume of EPSI spectra is lower than the SNR of spectra obtained through ordinary MRSI^4^.

The speed of EPSI can be fully utilized in high spectral and spatial resolution imaging of water and fat resonances in biological tissues^5–9^. Strong water and fat (-CH_2_-)n signals sufficiently compensate for SNR loss. High-resolution EPSI employs either a gradient echo train with symmetrical positive and negative readout gradient pulses^1^ or a flyback echo train, where readout gradients are combined with as short rewind gradients as possible^4,10–12^. The flyback approach necessitates stronger rewind gradients; however, data processing is simpler because all echoes are acquired with the same readout gradient polarity. A single gradient echo train typically has a narrow sBW due to gradient hardware limitations. This constraint can be addressed using multiple interleaved gradient-echo trains^2,5,13,14^.

A fundamental property of readout gradient echo trains is the inconsistency between echoes corresponding to positive and negative gradient pulses, as well as the inconsistency between interleaved gradient echo trains. The consequence is the appearance of Nyquist ghost spectral lines in the reconstructed spectra. This phenomenon reduces the intensity of each true spectral line due to energy transfer into its ghost peak(s). So far, methods for ghost peak reductions have been described exclusively for processing odd and even echoes of a single symmetrical gradient echo train^15–18^. The simplest method was proposed by Posse et al.^15^. The spectra from odd and even echoes were reconstructed separately and then averaged. This approach eliminates the ghost peaks for the cost of sBW reduction by a factor of two. The full sBW can be achieved by combining the odd and even echoes. The interlaced Fourier transform^16^, Fourier shift^17^, and temporal shift of the echoes, together with phase changes^18^, were proposed for this purpose. To our knowledge, no such procedure has been described for suppressing the ghost peaks produced by interleaved flyback echo trains.

The present study continues our previous work, in which we demonstrated and quantified the ghost spectral lines caused by two, three, and four interleaved flyback gradient echo trains^19^. This study aims to simulate ghost spectral lines produced by interleaved two flyback echo trains and to describe methods for reducing ghost peaks generated by two and four interleaved flyback gradient echo trains.

## Methods

### Animal models

Sprague Dawley rat was obtained from Taconic Biosciences, Lille Skensved, Denmark. Experiments were conducted in accordance with European Union Directive 2010/63/EU for animal research. The study received approval from the Ethical Committee for Animal Research of the Uppsala Region (permit number: 5.8.18–13038/2019). All animal procedures adhered to ARRIVE guidelines and followed the Uppsala University guidelines on animal experimentation (UFV 2007/724). The Sprague Dawley rat, twelve weeks old, and weighing ~ 327 g was kept sedated by gas anesthesia (Sevoflurane) during the introduction scans. Breathing rate was monitored using a commercial air pillow probe. Body temperature was preserved at 37 °C by warm air. The rat was sacrificed by increasing the concentration of Sevoflurane.

### Data acquisition

**Fig. 1:**
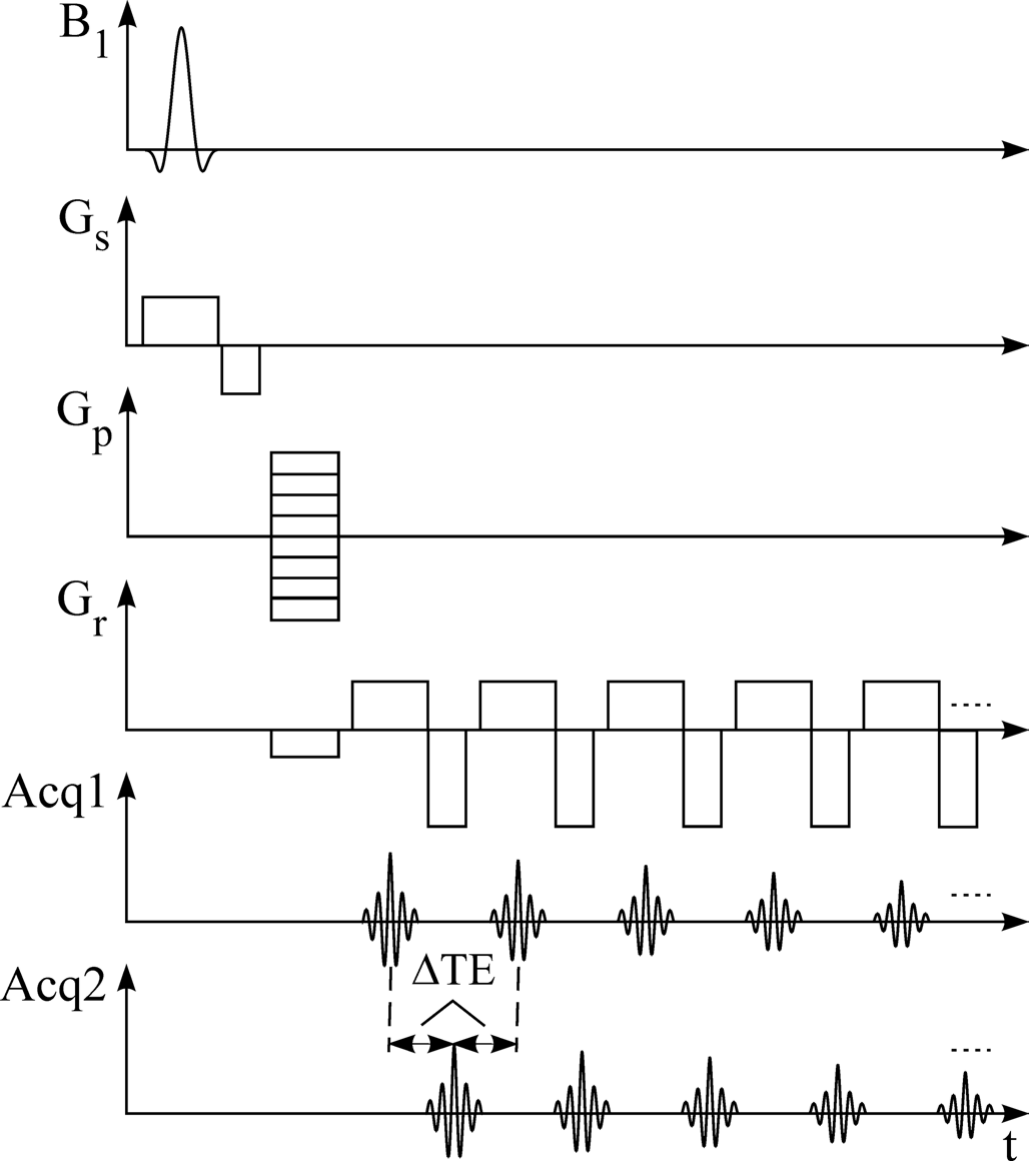
The high-resolution echo-planar spectroscopic imaging sequence with flyback readout. Acq1 shows a single gradient echo train. Data for interleaved two echo trains were acquired by combining echo train Acq1 and ΔTE-shifted echo train Acq2.

A multislice 2D flyback EPSI sequence was implemented in a 3 Tesla PET-MRI preclinical animal scanner (nanoScan, Mediso Medical Imaging Systems, Budapest, Hungary) equipped with 550 mT/m gradients and 4500 T/m/s slew rate. Selective RF excitation was followed by phase encoding and a trapezoidal flyback gradient echo train with 0.15 ms ramps (Fig. 1). The minimum length of rewind gradients was 0.6 ms (including ramps). First-order shimming was applied before each acquisition. Muscle and fat structures were simulated with the cylindrical phantom (inner diameter 26 mm, length 110 mm) containing a water solution of MnCl_3_ (0.23 mM, T_2_ ~ 30 ms)^20^ and vegetable oil (T_2_ ~ 70 ms). All measurements were performed with a field of view (FOV) of 80 × 80 mm, 192 phase-encoding steps, and 256 echo points (in-plane resolution of 0.31 × 0.42 mm²).

The acquisition with one flyback echo train was performed to acquire raw data for ghost spectral lines simulations, as spectra reconstructed from a single echo train do not contain ghost peaks^11,19^. The following scan parameters were used for the water-oil phantom: slice thickness 1.5 mm, 2 averages, TR/TE_1_ 200/3 ms, flip angle 35°, 96 echoes, echo spacing ΔTE 1.55 ms (sBW 5.03 ppm), and rBW 434 028 Hz (readout gradient G_r_ 127.42 mT/m).

Measurements with two and four interleaved flyback echo trains were conducted to demonstrate the reduction of ghost peaks. A water-oil phantom and a rat were used for this purpose. Acquisitions with interleaved two echo trains contained 48 echoes in each train. Experiments with interleaved four echo trains used 24 echoes in each train. Measurement parameters were identical (slice thickness 1.5 mm, 2 averages, TR/TE_1_ 200/3 ms, flip angle 45°, echo spacing ΔTE 0.8 ms (sBW 9.74 ppm), rBW 434 028 Hz. The rat was measured with interleaved two echo trains a few minutes after sacrifice.

### Data processing

The processing of measured raw data was performed using in-house-developed software. Data processing of the flyback raw data has been described elsewhere^19^. Spectrum processing consists of the following main steps: (i) zero-filling the input raw data matrix (k_read_, k_phase_, k_t_) to the matrix (256, 256, 256); (ii) fast Fourier transform (FFT) in the k_t_ dimension; (iii) removing chemical shift artifacts by the first-order phase correction in the spectral domain w_t_; (iv) performing a 2D FFT along k_read_ and k_phase_ dimensions; (v) correcting voxel spectral shifts caused by static magnetic field B_0_ deviations; (vi) calculating water and fat images by integrating the water and fat (-CH_2_-)n spectral lines; (vii) summing the voxel’s module spectra from a defined volume of interest (VOI); (viii) processing the spectrum using the Magnetic Resonance User Interface (MRUI) software^21^.

### Ghost peak simulation

All simulations were conducted with raw data acquired by a single flyback gradient echo train. Simulations were performed for interleaved two echo trains. The measured 2D echo matrices (k_read_, k_phase_) were reorganized into two echo trains before data processing. The interleaved second echo train contained even echoes of the original flyback echo train. The goal of the simulations was to demonstrate the impact of temporal shifts, magnitude, and phase changes of echoes in the interleaved second echo train on the formation of ghost peaks. The temporal offset of echoes in the second echo train was performed via cubic spline interpolation. All echoes in the second echo train were shifted in the readout direction by ± 0.5, ± 1, and ± 1.5 echo points, corresponding to a temporal offset of ± 1.15, ± 2.3, and ± 3.46 µs, respectively. Magnitude changes of echoes in the second echo train were achieved by multiplying echoes by constants 0.7, 0.8, 0.9, 1.1, 1.2, and 1.3. The last simulation involved zero-order phase shifts (Φ_0_) of all echo points in the second echo train by ± 20°, ± 40°, and ± 60°.

### Ghost peak suppression

Zero-order phase correction was applied to all echo points in the considered echo train to reduce ghost peaks. For interleaved two echo trains, correction was carried out only for the second train to optimize the phase to be consistent with the first echo train. Zero-order phase correction Φ_0_ was determined through an iterative procedure aimed at minimizing the ratio of the magnitude of the ghost peak to the magnitude of the true peak (GTR)^18^. Phase Φ_0_ was gradually increased from 0° until the minimum GTR was found. For interleaved four echo trains, zero-order phase corrections were applied to the second, third, and fourth echo trains. GTR was calculated as the ratio of the average magnitude of all ghost spectral lines to the magnitude of their true peak. It was assumed that zero-order phase correction Φ_0_ increases with step DF for the third and fourth echo trains. The first iteration was performed with the identical Φ_0_ for the second, third, and fourth echo trains. The second iteration focused on optimizing the phase difference ΔΦ. The phase correction can be determined using a water phantom. Resulting phase constants Φ_0_, and ΔΦ can be applied to all measurements performed with the same sequence and acquisition parameters.

**Fig. 2:**
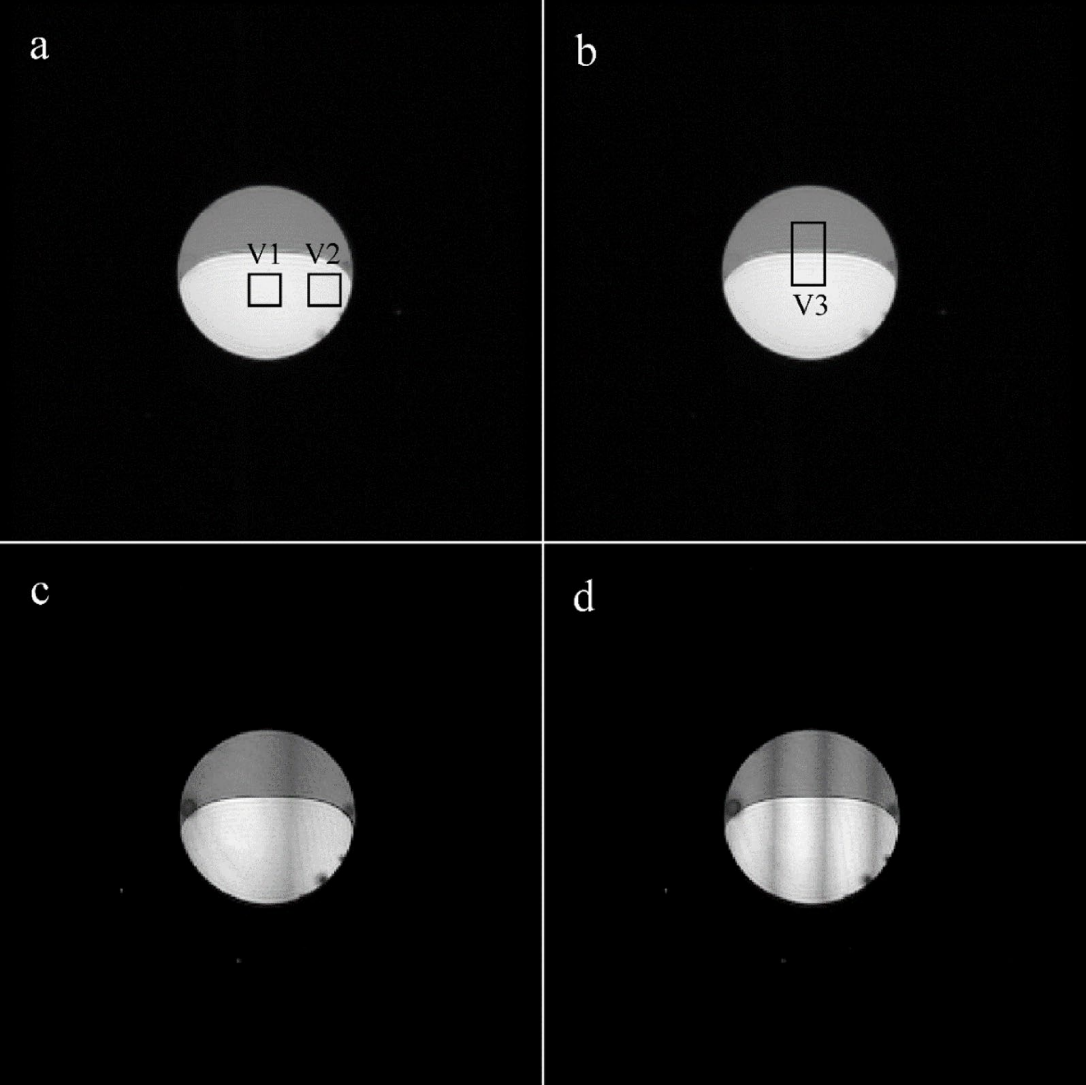
Chemical shift artifact-free images of the phantom contained vegetable oil (top) and water. The intensity of the images is proportional to the peak heights of the highest voxel spectral line. The acquisition was performed by interleaving two flyback gradient echo trains. (**a**) VOI V1 and V2 included 225 water voxels (43.2 mm^3^), and (**b**) V3 included 450 water and oil voxels (86.5 mm^3^). (**c**) The dark vertical strip was caused by the temporal shift of echoes by 2.5 echo points (5.76 µs) in the second echo train. (**d**) Three dark strips formed due to the time shift of echoes by 5.5 echo points (12.67 µs) in the second echo train.

## Results

**Fig. 3:**
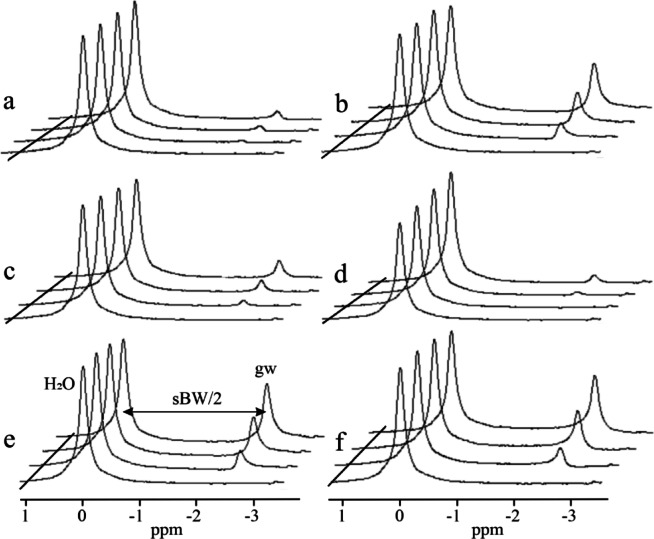
Simulation of ghost peak for interleaved two flyback gradient echo trains. The true water line was set at 0 ppm. The reference spectrum, without a ghost peak in each group of the spectra, was computed from the original flyback echo train. (**a**, **b**) Simulations of the water ghost peaks, gw, caused by the temporal offset of echoes in the second echo train by 1.1, 2.3, and 3.46 µs. Spectra were computed from the VOI (**a**) V1, and (**b**) V2 shown in Fig. 2. (**c**, **d**) Simulations of the ghost peaks induced by changes in the magnitude of echoes in the second echo train. (**c**) Echoes decreased by factors of 0.9, 0.8, and 0.7. (**d**) Echoes increased by factors of 1.1, 1.2, and 1.3. (**e**, **f**) Simulation of ghost peaks caused by zero-order phase shifts of echoes in the second echo train by (**e**) -20°, -40°, -60°, and (**f**) by 20°, 40°, 60°.

**Fig. 4:**
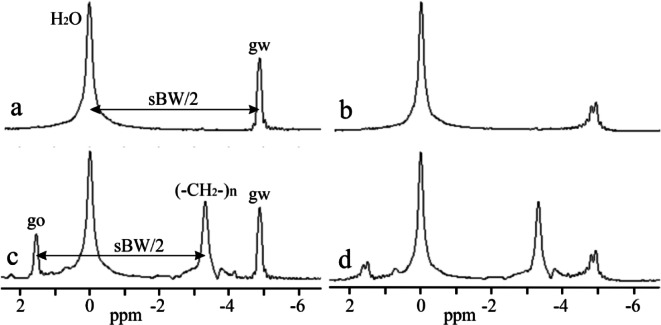
Water and vegetable oil spectra were acquired by interleaving two echo trains. The true water line was placed at 0 ppm. (**a**, **b**) True water line and its ghost peak, gw, before (**a**) and after (**b**) ghost peak suppression. Spectra were computed from VOI V1 (Fig. 2). (**c**, **d**) True water and oil (-CH_2_-)n spectral lines and their ghost peaks, gw, and go, before (**c**) and after (**d**) ghost peak suppressions. Spectra were computed from VOI V3 (Fig. 2).

**Fig. 5:**
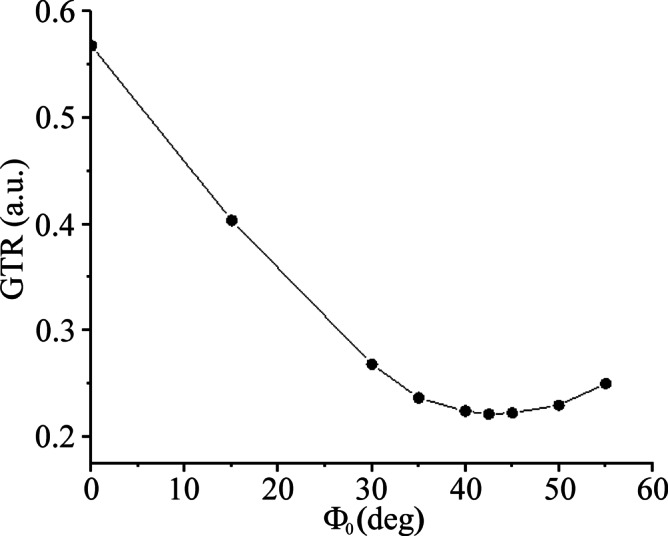
The ratio of the magnitude of the water ghost peak to the magnitude of the true water peak (GTR) as a function of zero-order phase correction Φ_0_. Water spectra were acquired by interleaving two echo trains (Fig. 4a,b).

**Fig. 6:**
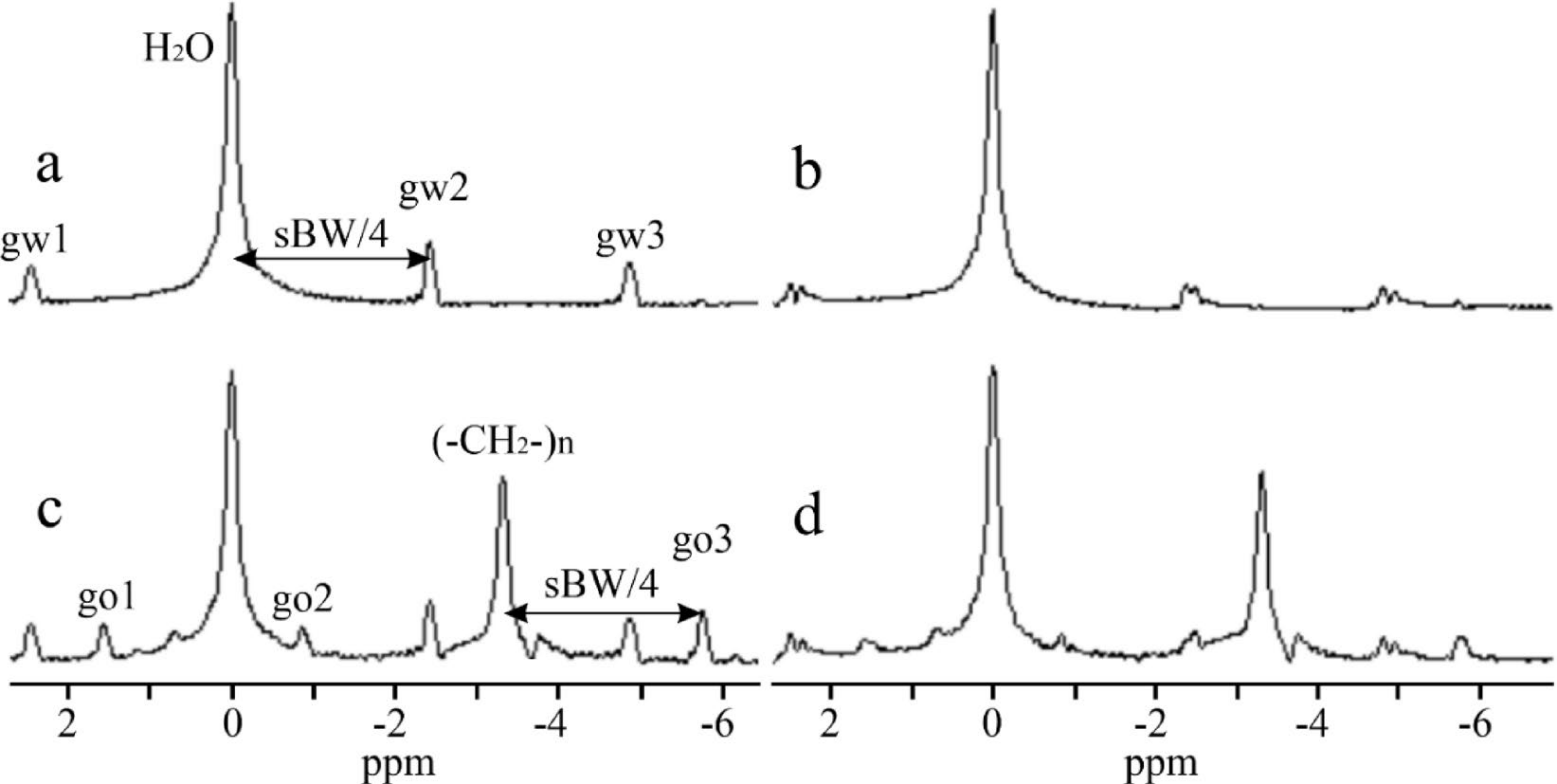
Water and vegetable oil spectra were measured by interleaving four echo trains. The true water line was set at 0 ppm. (**a**, **b**) True water line and its ghost peaks, gw1, gw2, and gw3, before (**a**) and after (**b**) ghost peak suppression. Spectra were computed from voxel V1 (Fig. 2). (**c**, **d**) True water and oil (-CH_2_-)n spectral lines and their ghost peaks, gw1, gw2, gw3, and go1, go2, go3, before (**c**) and after (**d**) ghost peak suppression. Spectra were computed from voxel V3 (Fig. 2).

**Fig. 7:**
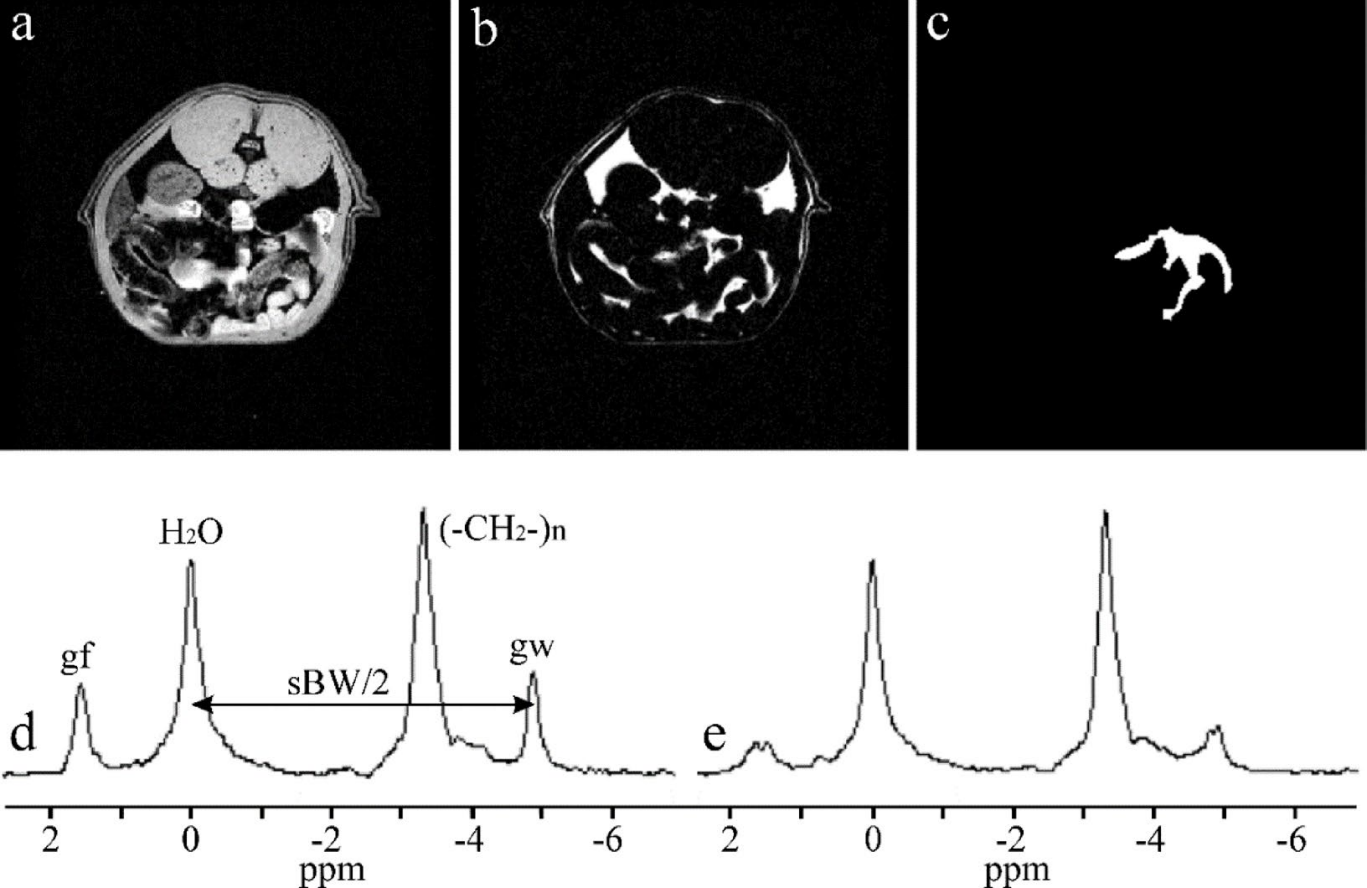
Water-fat images and spectrum acquired by interleaving two echo trains. (**a**) Transversal water and (**b**) fat image of the rat’s abdomen. (**c**) Spectroscopic VOI. (**d**) Original spectrum with true water, fat (-CH_2_-)n spectral lines, and their ghost water (gw) and fat (gf) peaks. (**e**) Spectrum after ghost peak suppressions.

Ghost peak simulations were performed for interleaved two-flyback echo trains using the water spectral line computed from VOI V1 and V2, as shown in Fig. 2. Figures 3a and b demonstrate the formation of ghost peaks caused by the temporal offset of echoes. The echoes of the second echo train were shifted concerning the first echo train by 1.1, 2.3, and 3.46 µs in the readout direction. A temporal offset in the opposite direction (-1.15, -2.3, -3.36 µs) had an identical effect on the ghost peak amplitudes. The magnitude of the ghost peak increases with the temporal offset of the echoes and with the distance of the voxel from the center of the magnet in the readout direction. Consequently, the magnitude of the ghost peak computed from VOI V2 (Fig. 3b) was larger compared to the ghost peak acquired from the central VOI V1 (Fig. 3a). However, the magnitude of the ghost peak did not vary with the distance of the voxel from the center of the magnet in the phase-encoding direction. If the temporal offset increased to about 2 echo points (~ 2.6 µs), then the magnitude of the ghost peak became comparable with its true spectral line, and a dark strip perpendicular to the readout direction appeared in the images of the peak heights of the highest spectral line in the voxel (Fig. 2c). The number of dark strips increased with increasing temporal offset (Fig. 2d). The alternating bright stripes originate from alternating highest true and ghost peaks. Figures 3c and d show ghost peaks caused by the magnitude changes of echoes in the second echo train. The rise of the ghost peak caused by zero-order phase shifts is shown in Fig. 3e and f. Figure 4 presents water and vegetable oil spectra before and after ghost peak suppression. The spectra were acquired using interleaved two echo trains. The GTR was minimized with a zero-order phase correction of 42.5° applied to all echo points in the second echo train (Fig. 5). The GTR of the water and oil (-CH_2_-)n spectral line decreased by a factor of 2.4 (Fig. 4b,d). Accordingly, the magnitude of the true peaks increased. Water and vegetable oil spectra measured by interleaved four echo trains are shown in Fig. 6. The lowest GTR was achieved by applying zero-order phase corrections of -25^o^, -28.5^o^, and − 32^o^ for the second, third, and fourth echo trains, respectively. The GTR of water and oil (-CH_2_-)n spectral lines decreased by a factor of 2.2. Figure 7 illustrates the application of the proposed phase correction to the rat’s spectrum. The measurement was performed by interleaving two echo trains a few minutes after the rat’s death. The VOI, which contained water and visceral fat (Fig. 7c), was created manually using the fat image (Fig. 7b).

## Discussion

To our knowledge, this is the first work that systematically simulates the ghost peaks in EPSI. Our simulations leveraged the fact that a single flyback echo train does not produce ghost spectral lines^11,19^. The insights gained from the simulations informed the development of methods for reducing ghost peaks. Experiments were conducted on a preclinical magnetic resonance (MR) scanner.

Magnitude and phase discontinuities between successive echoes cause ghost spectral lines in “conventional” EPSI with symmetrical gradient echo trains, as well as in the interleaved flyback EPSI. These discontinuities may originate from several factors, such as system timing errors, eddy currents, imperfect gradient waveforms, magnetic susceptibility artifacts, and static magnetic field inhomogeneities^12,16,18,22^, etc. Our simulations were performed for interleaved two flyback gradient echo trains. The simulations aimed to demonstrate the impact of temporal echo shifts, magnitude, and phase changes of echoes in the second flyback echo train on the formation of ghost spectral lines.

The effect of system timing errors was simulated by introducing a time shift of the echoes in the second gradient echo train. Temporal shift produces a ghost peak, and its magnitude increases with the distance of the voxel from the center of the magnet in the readout direction (Fig. 3a,b), but not in the phase-encoding direction. It should be noted that this dependence of ghost peak magnitude on voxel position is common for both conventional EPSI and flyback EPSI^18^. Temporal shifts in the echoes of the second echo train did not enhance ghost peak suppression. The ghost peak magnitude increased with a temporal offset of echoes. We conclude that the time shift of the flyback echo train caused by system timing errors can be excluded. This finding aligns with the study of Cunningham et al.^11^, who demonstrated the robustness of interleaved flyback EPSI to system timing errors and consequently to eddy currents. Time shifts greater than ~ 2 echo points (4.6 µs) in the second echo train produce vertical dark strips in the images of the peak heights of the highest spectral line in the voxel (Fig. 2c,d). We note that the same dark strips appear in uncorrected images acquired by conventional EPSI^18^.

Magnitude discontinuity between echo trains was simulated by multiplying echoes in the second echo train by a constant. Both increasing and decreasing echo amplitudes produced ghost peaks (Fig. 3c,d). However, an unrealistically large change in echo amplitudes was needed to generate a detectable ghost peak. From this fact, it follows that amplitude changes of echoes caused by the scanner’s hardware are unlikely.

Zero-order phase shifts were identified as the most significant reason for ghost peak formations, thanks to simulations (Fig. 3e,f). Therefore, zero-order phase corrections were employed for ghost peaks suppression (Figs. 4, 6 and 7). While optimizing the phase correction for interleaved two echo trains was straightforward, optimizing for four echo trains proved more complicated. Individually optimizing the zero-order phase correction for the second, third, and fourth echo trains did not yield the best ghost peak suppressions. The zero-order phase correction of only the second echo train reduced the amplitude of the ghost peak gw1. Still, peaks gw2 and gw3 increased (Fig. 6). The phase correction of the third echo train improved the suppression of ghost peaks gw1 and gw2, but the magnitude of peak gw3 increased. Conversely, the individual phase correction of the fourth echo train decreased ghost peak gw3, but at the cost of an increase in ghost peaks gw2 and gw3. The advantage of the proposed phase corrections is that Φ_0_ and ΔΦ constants can be optimized using a water phantom and applied to all measurements performed with the same sequence and acquisition parameters.

Despite its simplicity, the proposed zero-order phase corrections significantly reduced the magnitudes of ghost peaks while increasing the intensities of the true peaks. However, these corrections could not eliminate ghost peaks, as the inconsistencies between interleaved echo trains may stem from other factors as well, such as the spatial distribution of static magnetic field inhomogeneities (background gradients), nonideal gradient waveforms, magnetic susceptibility artifacts, etc. These issues may introduce complex nonlinear phase errors^22^. Addressing these problems requires sophisticated spectrum reconstruction.

The applicability of the flyback EPSI was already demonstrated on a clinical 3T scanner. The flyback readout was implemented in a PRESS pulse sequence and utilized for 3D ^1^H-MRSI of the human brain and prostate^11,12^. The spectral bandwidth 7.6 ppm was achieved with a single echo train. The spatial resolution and ghost-free spectra were comparable to those of conventional MRSI. However, the high spatial resolution requires two or more interleaved echo trains. The high-resolution EPSI with flyback readout may be feasible for water-fat imaging and spectroscopy on 3T clinical scanners equipped with powerful gradients (> 100 mT/m) and a broad receiver bandwidth (> 400 kHz).

The main disadvantage of the flyback EPSI approach is a relatively narrow sBW, which is limited by the rewind gradient strength and a reasonable gradient slew rate to avoid peripheral nerve stimulation. We have demonstrated that a sufficient sBW (9.74 ppm) can be achieved with a moderate slew rate (0.15 ms ramps) and the powerful gradients available in preclinical MR scanners with a small bore size.

## Conclusion

In this study, we have demonstrated the simulation of ghost spectral lines for high-resolution EPSI with flyback readout. The knowledge gained from simulations has been applied to propose methods for suppressing ghost peaks caused by two- and four-flyback gradient echo trains. Zero-order phase correction was identified as the most effective method to resolve inconsistencies between echo trains. Results obtained from phantom and rat experiments demonstrate that the described zero-order phase corrections significantly reduced ghost peak magnitudes while increasing the intensities of true peaks. The proposed ghost peak suppression approaches are suitable for water-fat spectroscopy and imaging with high spatial and spectral resolution.

## Data Availability

The datasets acquired and/or analyzed during the current study are available from the corresponding author on reasonable request.
